# A Case Report of Intussusception After Gastric Bypass

**DOI:** 10.7759/cureus.10459

**Published:** 2020-09-15

**Authors:** Rafik Guerroudj, Sofia Takkal, Bernard Hainaux

**Affiliations:** 1 Radiology, Saint Pierre Hospital, Brussels, BEL; 2 Radiology, Saint Pierre Hospital, Brussels , BEL

**Keywords:** by pass, intussusception

## Abstract

Intussusception is a rare cause of late complication after gastric bypass. We report the case of a 53-year-old woman having a gastric bypass in 2011. The patient presented to the emergency department with abdominal pain and vomiting. The diagnosis of intussusception was made by CT scan. Laparoscopy found an invaginated intestinal segment at the level of the jejuno-jejunal anastomosis without necrosis. Adhesiolysis and revision of the anastomosis were performed. The post-operative course was favorable. The diagnosis of intussusception was made by CT scan.

## Introduction

Bariatric surgery has become a common surgical technique for the treatment of morbid obesity worldwide, it is more effective compared to conservative treatment with greater weight loss and lower morbidity related to obesity. Among the many surgical techniques, the most common procedure in the world is the gastric bypass [1].

Intussusception is rare in adults (only 5%), almost 90% of intussusception in adults are secondary to a pathologic lead points (polyps, inflammatory process or tumors) [2].

Like any surgery, gastric bypass can have complications that sometimes require revision. The most common long-term complication is bowel obstruction due to internal hernia or adhesions. Another less common complication is intussusception, which can occur many years after surgery [3].

The diagnosis of intussusception is difficult because the symptoms and laboratory results are not specific. The classic triad of abdominal pain, bloody stools and a palpable mass is rarely seen. The symptoms may be acute, chronic, or intermittent [4].

## Case presentation

A 53-year-old woman presented to the emergency department with diffuse non-specific abdominal pain for the last three weeks associated with vomiting. The patient also reported asthenia and a weight loss. The patient underwent a gastric bypass in 2011.

**Figure 1:**
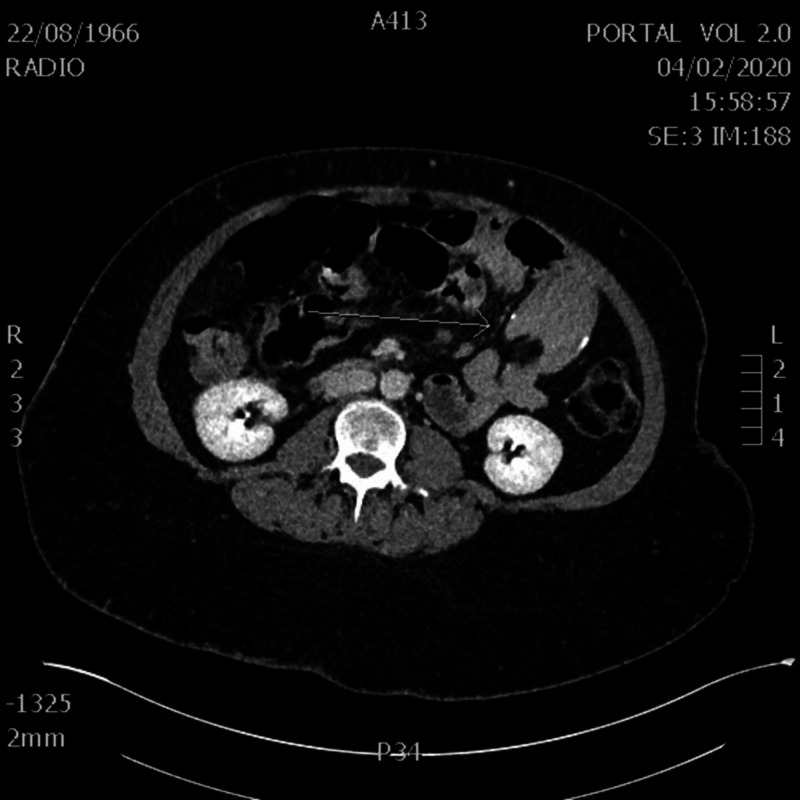
Axial view of the CT scan showing intussusception at level of jejuno-jejunal anastomosis

**Figure 2:**
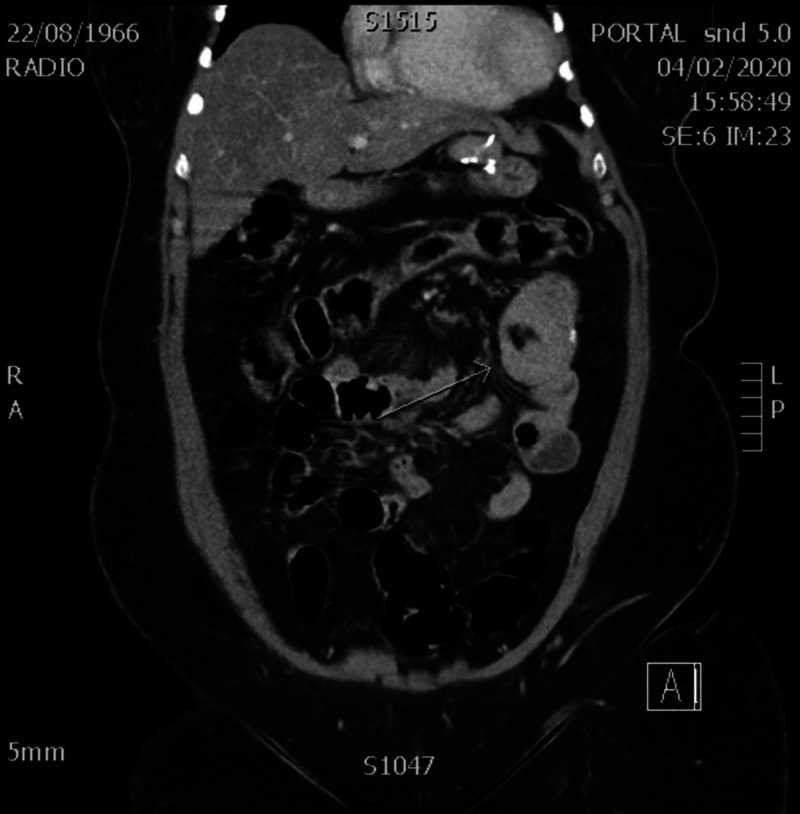
Coronal view of the CT scan showing intussusception at level of jejuno-jejunal anastomosis

The physical examination revealed hypertympanic distended abdomen and hypochondrium mass at palpation. Blood test was unremarkable. The abdominal CT scan showed an intussusception at the level of the jejuno-jejunal anastomosis in the left hypochondrium. The CT also revealed an acute obstruction of the proximal jejunum with segmental dilatation and stasis. These findings were confirmed at laparoscopy performed 15 hours after the CT. Laparoscopic exploration revealed dilated small bowel loops around the jejuno-jejunal anastomosis and intussusceptions on a fibrous adhesion encircling the jejunum proximaly to the common channel. Intussusception reduction was achieved and the post-operative course was favorable (Figures 1, 2).

## Discussion

Intussusception is a rare cause of obstruction after gastric bypass, its incidence is 0.1% to 1.2% with a median interval of 36-52 months after the procedure [4].

Intussusception is defined as the pull of a segment of the gastrointestinal tract into an adjacent segment, the exact mechanism is unclear, but an increase of peristalsis and the caliber of the small bowel is cited in the literature. Intussusception occurs most frequently at the anastomotic site of the proximal jejunum [5].

The classic triad of abdominal pain, bloody stools, and a palpable mass is rarely seen in these cases of intussusception, only 10% of patients have this triad. The majority of patients presented with nonspecific abdominal symptoms including diffuse acute, chronic, or intermittent abdominal pain, nausea, and vomiting [2].

Ultrasound is the technique of choice for children. In adults, CT with contrast injection is the technique of choice and the most sensitive technique, he does the diagnosis, give the location (small or large bowel intussusception), the cause (benign, malignant, or idiopathic), and look for complications.

Three CT signs are described in intussusceptions in relation to the evolution of the pathology: the "target sign" and “sausage-shaped” mass, representing bowel-within-bowel with alternating layers of low (mesenteric fat) and high (bowel wall) attenuation forming concentric rings when imaged at right angles to the lumen, and a soft tissue sausage when imaged longitudinally. And at the end, a “kidney mass” formed by the mesenteric fat, dragged into the intussusception mimicking the renal hilum, and by the bowel edema mimicking the renal parenchyma.

Abdominal ultrasound and X-ray have no place in the diagnosis in adults [6].

As the symptoms are not very specific and are similar to other post-operative complications like internal hernia or bridles, the CT scan should be performed in emergency in all operated patients who have acute or chronic abdominal pain of unknown origin. Emergency laparoscopy should be performed for all patients who have signs of peritonitis or shock [7].

Early laparoscopic surgical exploration is recommended to prevent progression to infarction and perforation, it reduces morbidity and mortality of patients. The surgical technique consists of revision alone in the absence of ischemia or resection with revision of the anastomosis if ischemia is present [4].

## Conclusions

The diagnosis of intussusception in adults is relatively rare and are secondary to a pathologic lead points, it can also be a rare long-term complication of gastric bypass with serious consequences like small bowel ischemia or perforation. The etiology is not very well understood but most of the authors suggest a disturbance in the motility of the digestive loops following surgery. Diagnosis is difficult because the symptoms and laboratory results are non-specific, the majority of patients presented with acute, chronic, or intermittent abdominal pain, nausea, and vomiting. Internal hernia should always be included in the differential diagnosis. Abdominal ultrasound and X-ray have no place in the diagnosis. The scanner is the technique of choice for diagnosis and to look for complications, it must be performed in all patients operated with abdominal pain. Early surgery with reduction of the intussusception, revision of anastomosis and bowel resection of non-viable bowel reduces morbidity and mortality.
